# Virulence-Associated Genes of *Calonectria ilicola*, Responsible for *Cylindrocladium* Black Rot

**DOI:** 10.3390/jof8080869

**Published:** 2022-08-18

**Authors:** Xinyu Chen, Mei Luo, Wei Wu, Zhangyong Dong, Huasong Zou

**Affiliations:** 1 Innovative Institute for Plant Health, Zhongkai University of Agriculture and Engineering, Guangzhou 510225, China; 2 College of Plant Protection, Fujian Agriculture and Forestry University, Fuzhou 350002, China; 3 Key Laboratory of Green Prevention and Control on Fruits and Vegetables in South China, Ministry of Agriculture and Rural Affairs, Zhongkai University of Agriculture and Engineering, Guangzhou 510225, China

**Keywords:** *Cylindrocladium* peanut black rot, *Calonectria ilicicola*, genome, transcriptome, proteome, virulence-associated gene

## Abstract

The *Cylindrocladium* black rot caused by *Calonectria ilicicola* is a destructive disease affecting a broad range of crops. Herein, we study virulence-associated genes of *C. ilicicola* *Ci*14017 isolated from diseased peanut roots (*Arachis hypogaea* L.). *Ci*14017 was identified via phylogenetic analysis of the internal transcribed spacer region and standard Koch’s postulate testing. Virulence-associated genes were based on genome analyses and comparative analysis of transcriptome and proteome profiles of sensitive and resistant peanut cultivars. *Ci*14017 identified as *C. ilicicola* has a 66 Mb chromosome with 18,366 predicted protein-coding genes. Overall, 46 virulence-associated genes with enhanced expression levels in the sensitive cultivars were identified. Sequence analysis indicated that the 46 gene products included two merops proteins, eight carbohydrate-active enzymes, seven cytochrome P450 enzymes, eight lipases, and 20 proteins with multi-conserved enzyme domains. The results indicate a complex infection mechanism employed by *Ci*14017 for causing *Cylindrocladium* black rot in peanuts.

## 1. Introduction

*Cylindrocladium* black rot (CBR) in peanuts is a destructive fungal disease caused by *Calonectria ilicicola* (anamorph: *Cylindrocladium parasiticum*) [1,2]. The associated black and rot symptoms develop on peanut (*Arachis hypogaea* L.) needles, pods, and roots, eventually leading to wilting of the whole plant [3]. CBR was first discovered in Georgia, USA in 1965 [1], and has spread to various peanut production areas, including the southeastern United States, Japan, India, and Australia [1,2,4,5]. In China, the *C. ilicicola* pathogen has been reported to cause disease in several host plants, including *Zingiber officinale* Rosc., *Medicago sativa* L., *Manglietia deciduas*, and *Vaccinium* spp. [6,7,8,9]. Owing to the potential threat it poses, *C. ilicicola* was listed in the “List of Entry Phytosanitary Pests of the People’s Republic of China” in 2007 [10].

*Calonectria ilicicola* is a soil-borne pathogen transmitted mainly through seeds to invade hosts, which best explains difficulties in effective prevention and control [11]. Soil fumigation with methyl isothiocyanate does suppress CBR occurrence in severely affected fields to a degree, but at too high a cost [12]. The discovery of resistance mechanisms has been difficult, as no highly resistant varieties have been introduced yet, with only some medium-resistant peanut varieties identified [13]. Therefore, understanding the detailed infection mechanisms of *C. ilicicola* will help explore additional approaches to control CBR disease.

Pathogenic fungi attack host plants through several strategies, utilizing adhesion to the plant surface, formation of a particular infection structure, surface breakthrough, colonization, and amplification *in planta* [14]. The fungi enter plant tissues through stomata or wounds via the haustorium, infection cushion, and the appressorium, a critical infection structure [15,16]. Plant fungi secrete various enzymes that act as cell wall degradation enzymes, toxins, or growth regulatory substances for optimal disease development [17,18,19]. Furthermore, fungi release a repertoire of effector proteins into host cells, helping to modify host defenses and promote susceptibility [20]. These effectors are defined, in a narrow sense, as secreted proteins of less than 300 amino acids and rich in cysteine [21]. They are further characterized by N-terminal signal peptides, no transmembrane domain, multiple repeating elements, and small-molecule proteins [22,23].

RNA and protein sequencing technologies are powerful tools for the discovery of functional fungal genes or proteins involved in virulence. For example, the genes involved in degradation enzyme activity and nucleic acid metabolism were found to be required for virulence through the comparison of transcriptome and proteome data in susceptible and resistant *Spongospora subterranea* hosts [24]. Although CBR has been reported in China for many years, little is known regarding the molecular pathogenic mechanisms employed by *C. ilicicola*. The purpose of this study was to identify candidate *C. ilicicola* virulence-related genes. Virulence-associated genes in *C. ilicicola* strain *Ci*14017 were screened on a susceptible peanut cultivar, P562, and a resistant cultivar, T09, via combined transcriptome and proteome analysis. The current results provide global knowledge on the putative pathogenic factors of *C. ilicicola*, which will add to our understanding of the genetic basis of CBR development.

## 2. Materials and Methods

### 2.1. Fungal Isolation

The pathogenic fungus was isolated from diseased peanut roots collected from Meizhou City, Guangdong Province, China. A standard tissue culture isolation method was performed on potato dextrose agar (PDA) for 7 days at 25 °C until fungus growth [25]. To purify the pathogen, six single fungal spores were transferred to a new PDA medium for hyphal growth. After 7 days of culture, the mycelia were selected to observe the micromorphological characteristics of the isolated strains under a light microscope and photographed for morphological identification [26]. After genomic sequencing and identification, the *C. ilicicola* strain *Ci*14017 was maintained on cryovials containing 25% glycerol and kept at −80 °C for long term storage and on PDA slope in 10–15 °C for short term storage. Both were stored at the Innovation Institute for Plant Health of Zhongkai University of Agriculture and Engineering.

### 2.2. ITS Sequencing and Phylogenetic Analysis

The genomic DNA of *Ci*14017 was extracted using the DNeasy Mini Kit (50, QIAGEN Hilden, Germany). The internal transcriber spacer (ITS) region was amplified via PCR using the primer pair ITS1 (5′-TTCCGAGGTGAACCTGCGG-3′) and ITS4 (5′-TCCTCCGCTTATTGATATGC-3′) [27]. PCR was performed as per the following protocol: initial denaturation at 94 °C for 2 min, followed by 30 cycles at 98 °C for 10 s, 50 °C for 30 s, 72 °C for 1 min, and a final extension at 72 °C for 10 min. The 561-bp purified PCR fragment was sequenced, and sequencing data were subjected to a BLASTn homology search against the NCBI nucleotide database (http://www.ncbi.nlm.nih.gov/), accessed on 27 April 2022. ITS nucleotide sequences from the *Ci*14017 isolate, 16 reference *Calonectria* species, and an outgroup strain of *Neonectria major* were used to establish a phylogenetic tree based on maximum likelihood, maximum parsimony, and MrBayes analyses with RaxML v2.0 [28], PAUP v4.0 [29], and MrBayes v3.2.6 [30], respectively.

### 2.3. Inoculation of Peanut Plants with C. ilicicola

Seeds of the peanut-sensitive cultivar P562 were immersed in 0.5 wt% sodium hypochlorite solution for 5 min for disinfection, then rinsed with sterile water three times. Twice the weight of seeds sterile water was added, and the peanut seeds were immersed for 12 h (25 ± 1 °C, in dark), rinsed with distilled water three times, and then sprouted in moist environment Petri dishes in an artificial climate chamber for germination (25 ± 1 °C, 70–100% humidity, in dark). Normally, peanut seeds germinate in two to three days in such conditions. Germinated seeds were cultivated in plastic plots (36 cm × 25 cm × 13 cm) containing sterile nutrient water (25 ± 1 °C, light/dark:16 h/8 h). The previous stages were carried out in a sterile environment. The *C. ilicicola Ci*14017 strain was cultured on potato dextrose (PD) for 5 days at 25 °C, and 1 g of fungal sample was resuspended in 100 mL sterile water (10^6^/mL conidial). Ten-day-old plants were inoculated with the conidial suspension. Control plants were inoculated with 100 mL sterile water. CBR symptoms were scored 9 days post-inoculation. To complete Koch’s postulate testing, *C. ilicicola* was re-isolated from diseased seedlings and subjected to ITS region analysis. Three replicates of the inoculation experiment were done.

### 2.4. Genome Sequencing and De Novo Assembly

Genome sequencing was carried out using an Illumina HiSeq 2000 Sequencer (Illumina/Solexa), generating 100-bp paired-end reads. Reads were de novo assembled using ABySS 1.5.2 (http://www.bcgsc.ca/platform/bioinfo/software/abyss), accessed on 18 April 2022, Velvet 1.2.1 (http://www.ebi.ac.uk/~zerbino/velvet), accessed on 21 April 2022, and SOAP denovo 2.04 (http://soap.genomics.org.cn/soapdenovo.html), accessed on 24 April 2022 with a k-mer setting of 31 to 97. The assembled genome was then assessed for completeness using BUSCO v3.0 [31] (Table S1).

### 2.5. Gene Prediction and Annotation

The interspersed repetitive elements of *Ci*14017 were determined and annotated with RepeatMasker version open-4.0.5 (http://www.repeatmasker.org), accessed on 26 April 2022, followed by rRNA and tRNA detection using RNAmmer v1.2 and tRNAscan-SE v1.3.1, respectively [32,33]. For the sequencing of coding genes, AUGUSTUS v3.3.3 (http://bioinf.uni-greifswald.de/augustus/binaries/), accessed on 27 April 2022 was used as a pretest software from the head, and *Fusarium graminearum* was used as a reference species for de novo gene prediction. The gene pool of BUSCO was selected from four database files of Ascomycota, fungi, Hypocreales, and Sordariomycetes. The predicted coding sequences were searched against the NCBI (http://www.ncbi.nlm.nih.gov/), accessed on 27 April 2022 and Swiss-Prot protein databases (http://www.uniprot.org/), accessed on 27 April 2022 using BLASTP, with an e-value of ≤1 × 10^−5^. The predicted coding sequences were analyzed using Blast2GO (http://www.geneontology.org/), accessed on 28 April 2022 for Gene Ontology (GO) annotation and Kyoto Encyclopedia of Genes and Genomes (KEGG) pathway mapping (http://www.genome.jp/kegg/), accessed on 28 April 2022. Functional classification of the predicted proteins was performed using KOG (http://ww.ncbi.nlm.nih.gov/KOG/), accessed on 28 April 2022, whereas fungal pathogenicity domains were determined using the MEROPS [34], DFVF [35] (http://sysbio.unl.edu/DFVF/), accessed on 28 April 2022, lipase [36], cytochrome P450 (CYP450) [37], pathogen–host interaction database (PHI-base) [38,39] (http://www.phi-base.org/downloadLink.htm), accessed on 28 April 2022, and CAZymes databases [40] (http://cricket.ornl.gov/cgi-bin/cat.cgi), accessed on 28 April 2022 via BLASTP, with an e-value ≤1 × 10^−5^.

Genomic collinearity analysis was performed on the whole genomes of *Ci*14017 and *F. graminearum* using MCScanX, with an e-value ≤ 1 × 10^−5^. The predicted genome models of *Ci*14017 and 18 fungal species were subjected to homologous gene analysis. A comparative analysis was performed using these data. All fungal genomes were selected based on the consanguinity strategy with the eight *Calonectria* species, including four isolates (*C. pseudonaviculata cb*12013, *C. pseudonaviculata cb*12034, *C. canadiana cc*13392, and *C. leucothoes cl*13394) from our laboratory and Professor Tom Hsiang’s team at the University of Guelph, Canada, which has not yet been published.

Putative secreted peptides were identified based on the presence of a signal peptide secretion signal, absence of a transmembrane domain, and localization within organelles. Secreted peptides and transmembrane domains were predicted using SignalP v4.1 [41] and TMHMM v2.0 [42] (http://www.cbs.dtu.dk/services/TMHMM/), accessed on 29 April 2022, respectively. Secreted location domains were predicted using TargetP v1.1 [43] (http://www.cbs.dtu.dk/services/TargetP/), accessed on 29 April 2022 and ProtComp v9.0 [42,43] (http://linux1.soft-berry.com/berry.phtml?topic=protcomppl&group=programs&subgroup=proloc), accessed on 29 April 2022. The potential effector proteins were characterized using the following criteria: (A) annotated in PHI-base; (B) implicated in reduced virulence, loss of pathogenicity, lethal, or hypervirulence status, as described by PHI-base; and (C) localized extracellularly (secreted protein).

### 2.6. RNA-Sequencing

To infect peanut plants with *Ci*14017, 1 g fungal sample resuspended in 100 mL sterile water (10^6^/mL) was inoculated on 10-day-old resistant cultivar T09 and the susceptible cultivar P562. At 9 days post-inoculation, root segments from the three treated seedlings were sampled. *Ci*14017 samples cultured in PDA medium were used as controls to monitor gene expression changes. For each sample, a pool was generated from three repeated collections. Total RNA was extracted from three sample pools using TRIzol reagent (Invitrogen, Carlsbad, CA, USA) according to the manufacturer’s instructions. To construct RNA-seq libraries, cDNA was synthesized by random hexamer primers using the short fragmented mRNA, which was purified by beads containing oligo (dT) and then fragmented using EB buffer. After amplification, the quality of the library was monitored using an Agilent Bioanalyzer 2100 system. After Illumina sequencing adaptors were ligated to the short fragments, sequencing was performed using an Illumina HiSeq^TM^ 2000 (100 bp, paired-end). In the quality control step, sample quality and identification were determined using an Agilent Bioanalyzer 2100 system (Agilent Technologies, Inc. La Jolla, CA, USA) and an ABI Step One Plus Real-Time PCR system (Applied Biosystems, Foster, CA, USA).

Clean reads were mapped to the reference genome (this study) using Bowtie2. FPKM values (expected number of fragments per kilobase of transcript sequence per million base pairs sequenced) of each unigene were calculated using RSEM (v1.3.0). Subsequently, the FPKM values were subjected to comparative analysis between sequencing groups with GFOLD to show relative expression levels [44]. Differences with an absolute fold change of FPKM value > 2 and q value ≤ 0.001 were considered statistically significant, and these unigenes were considered differentially expressed genes (DEGs).

### 2.7. Proteome Analysis

Proteins were extracted from three *Ci*14017 sample pools, as previously described [45]. Liquid chromatography/mass spectrometry (LC/MS) was performed to identify proteins showing differential expression levels between *Ci*14017 sample pools. The mobile phase consisted of 0.1% formic acid in water (A) and 0.1% formic acid in acetonitrile (B). The tryptic peptides were dissolved in 0.1% formic acid (solvent A) and then separated using a Bruker NanoElute (Germany) ultra-high performance liquid chromatography (UPLC) system. The gradient was comprised of an increase from 6% to 22% solvent B over 70 min, 22% to 32% in 14 min and climbing to 80% in 3 min, then holding at 80% for the last 3 min, all at a constant flow rate of 300 nL/min on UPLC system. The peptides were subjected to capillary ion source followed by tandem MS in Bruker timsTOF Pro (Germany) coupled online to the UPLC. The ion source voltage applied was 1.4 kV. The *m*/*z* scan range was 100 to 1700 for secondary MS. The data acquisition mode used parallel accumulation–serial fragmentation (PASEF) mode.

The resulting MS data were processed using Maxquant search engine (v.1.6.6.0). Tandem mass spectra were searched against the *Arachis hypogaea* L. (data not shown) and *Calonectria ilicicola* database (101,330 and 8978 proteins) concatenated with a reverse decoy database. Trypsin/P was specified as a cleavage enzyme allowing up to 2 missing cleavages. The mass tolerance for precursor ions was 70 ppm in the first search and 70 ppm in the main search, and the mass tolerance for fragment ions was 0.04 Da. False discovery rate (FDR) was adjusted to <1%. Proteins with a fold change (FC) greater than two were identified as differentially expressed.

### 2.8. Bioinformatic Analysis of Differentially Expressed Proteins (DEPs)

Multiple sequence alignment for every DEP was conducted using MAFFT (http://www.ebi.ac.uk/Tools/msa/mafft/), accessed on 2 May 2022 [46]. RaxML was used to construct a phylogenetic tree based on the maximum likelihood method [28]. Upset analysis was performed using the OmicStudio tool (http://www.omicstudio.cn/tool), accessed on 2 May 2022. The proteins were then subjected to GO annotation and InterProScan v5.14–53.0 (http://www.ebi.ac.uk/interpro/), accessed on 5 May 2022. Pathway involvement was determined using KEGG Mapper (http://www.kegg.jp/kegg/mapper.html), accessed on 5 May 2022. Heat maps were constructed and visualized using ChiPlot (http://www.chiplot.online/), accessed on 6 May 2022. The conserved domains were predicted using the Conserved Domain Database Structure in NCBI (http://www.ncbi.nlm.nih.gov/Structure/bwrpsb/bwrpsb.cgi?), accessed on 6 May 2022.

## 3. Results

### 3.1. Identification of the C. ilicicola Ci14017 Pathogen Isolated from Peanut

**Figure 1:**
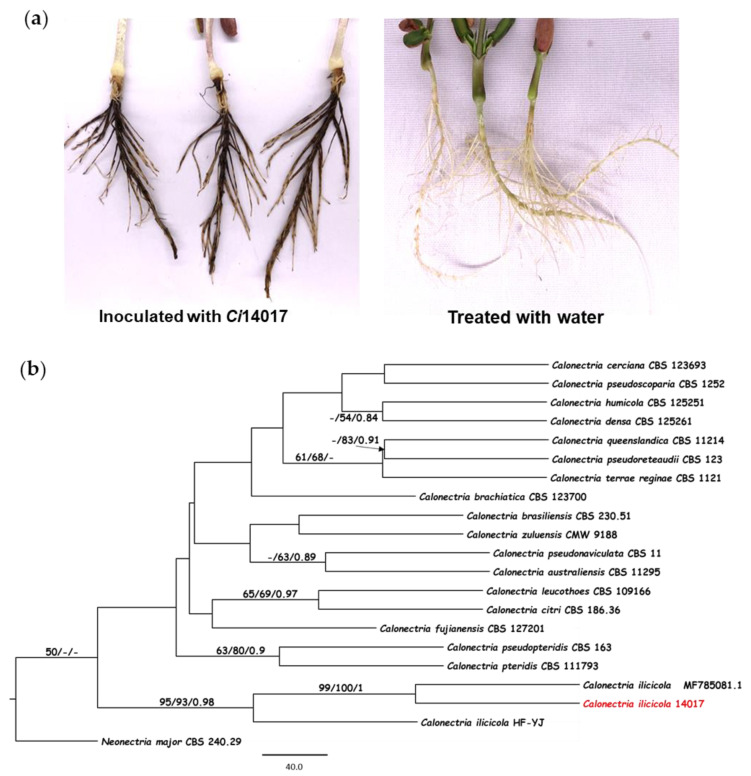
Isolation and identification of the *C. ilicicola Ci*14017. (**a**) The CBR symptoms on peanut roots inoculated with *Ci*14017. (**b**) Phylogenetic tree established from the ITS region of *Ci*14017. The phylogenetic tree based on Maximum likelihood, Maximum parsimony and MrBayes analysis, the bootstrap support rate of the three is merged into the ML evolutionary tree. The arrow in the figure points to the branch where the bootstrap support rate is located.

The CBR pathogen *Ci*14017 was isolated from diseased peanut roots as per a standard tissue culture isolation method. To verify the pathogenicity of *Ci*14017 in peanut plants, a 1% mycelium suspension was inoculated on 10-days-old susceptible cultivar P562. At 9 days post-inoculation, disfigured spots from brown to dark brown and necrotic rot appeared in the roots, with symptoms similar to those of diseased roots collected from the field (Figure 1a). Furthermore, the pathogen *Ci*14017 was re-isolated from symptomatic roots inoculated with mycelium suspension (data not shown). These results demonstrated that the *Ci*14017 isolate was the causal agent of peanut CBR.

Molecular identification of the *Ci*14017 isolate was performed via PCR amplification of the ITS gene region. The obtained 561-bp ITS1/4 DNA fragment was subjected to a homology search in the NCBI database. Fifteen homologs were found in *C. ilicicola*, sharing over 99% sequence identity. Three of these showed the highest identity of 99.29%, including the homologs from isolate HF-YJ (GenBank: MK990098.1), isolate PM-HHL-F018-WD (GenBank: MK850211.1), and an unnamed *C. ilicicola* isolate (GenBank: MF785081.1). The ITS sequences of *C. ilicicola Ci*14017, NA-LiaoNing2018, and HF-YJ, as well as those from 18 representative *Calonectria* species, were used to construct a phylogram. In the generated phylogenetic tree, *Neonectria major* strain CBS_240.29 fell into an out-group. Phylogenetic analysis indicated that *Ci*14017 falls into the cluster of *C. ilicicola* species, within the same clade as *C. ilicicola* MF785081.1 (Figure 1b).

### 3.2. Genomic Assembly and Features of C. ilicicola Ci14017

A total of 1993 scaffolds with an average length of 33,272 bp were generated from sequencing. These included the 40 smallest scaffolds of 121 bp and the largest scaffold of 1,935,007 bp. The assembled draft genome was 66,310,315 bp, with a GC content of 48.22%. The 10 largest scaffolds comprised 14,067,989 bp, accounting for 2.9% of the total genome length (Table 1).

### 3.3. Genome and Gene Functional Annotation

**Figure 2:**
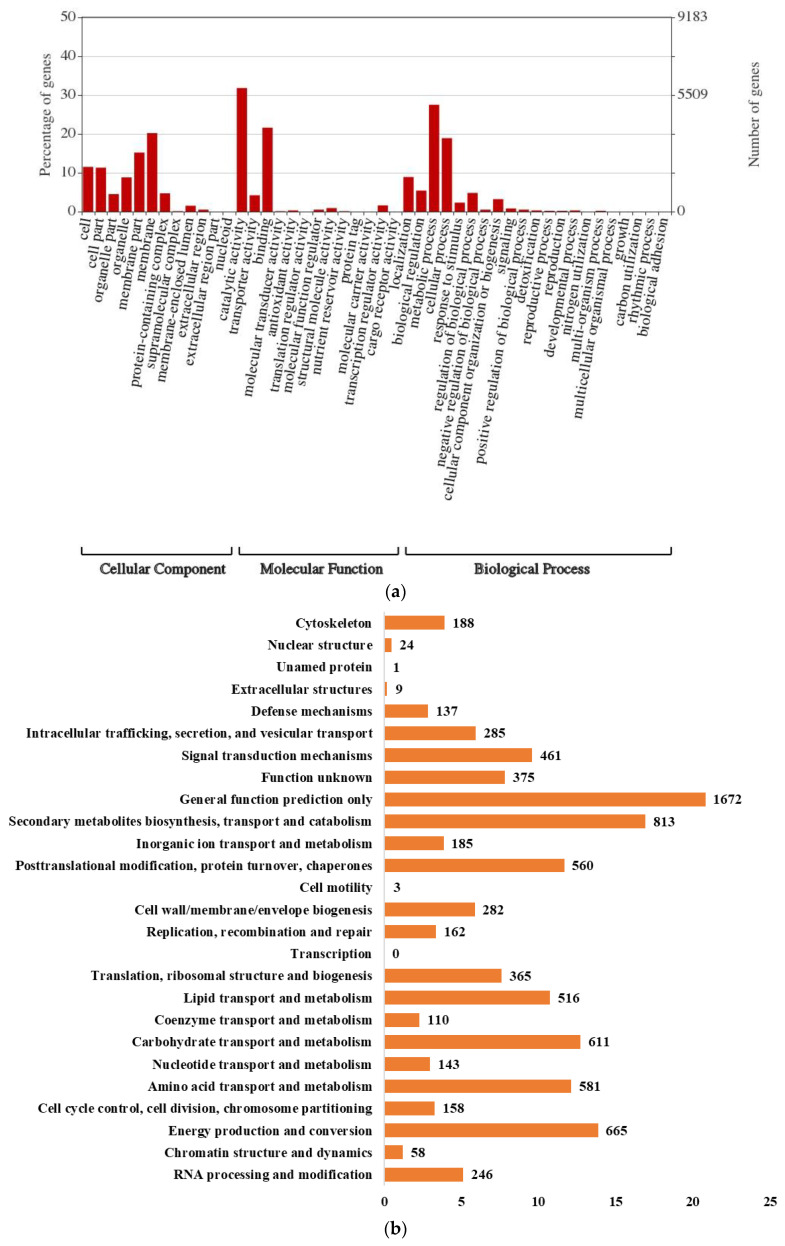

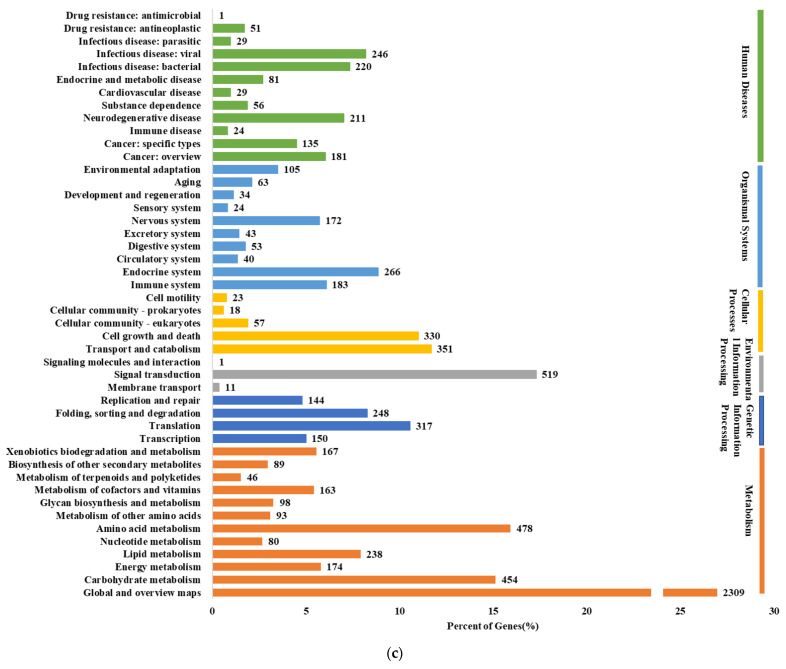
*Ci*14017 gene functions categories. (**a**) GO classification statistics of *Ci*14017. Blast2GO (http://www.geneontology.org/), accessed on 28 April 2022 for GO term annotation; (**b**) KOG classification statistics of *Ci*14017. KOG (http://ww.ncbi.nlm.nih.gov/KOG/), accessed on 28 April 2022; and (**c**) gene statistics of KEGG pathway in *Ci*14017. KEGG pathway mapping (http://www.genome.jp/kegg/), accessed on 28 April 2022.

The *Ci*14017 genome contained 18,366 coding genes with an average gene length of 1525 bp (Table 1). The gene density was three exons per gene, with an average of 522 bp. Ninety rRNAs and 423 tRNAs were predicted (Table 1). Functional annotation of coding genes in the *Ci*14017 genome was performed using the GO, KOG, and KEGG databases. Overall, 10,116 genes were annotated in the GO database, 8937 in the KOG database, and 8805 genes in the KEGG database (Figure 2a,b). Approximately 46.7% of these were categorized into five KEGG pathways: global and overview maps (2309), signal transduction (519), amino acid metabolism (478), carbohydrate metabolism (454), and transport and catabolism (351) (Figure 2c).

### 3.4. Comparative Genomics Analysis

**Figure 3:**
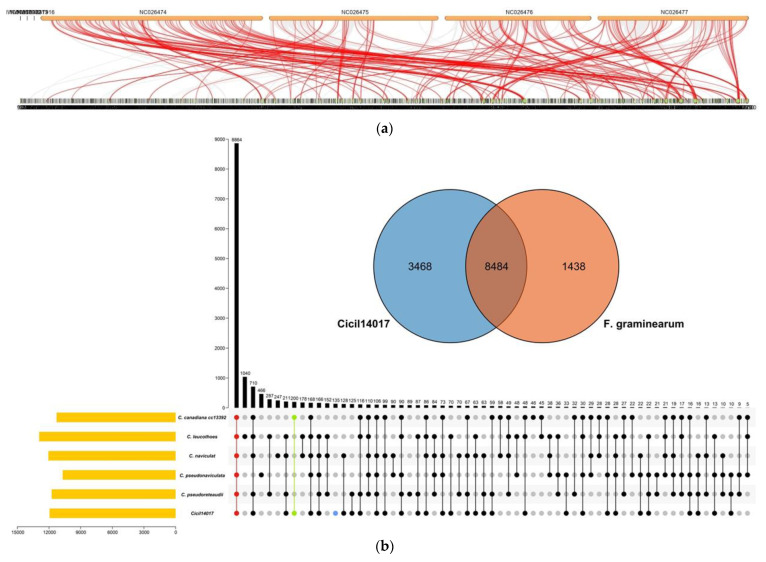

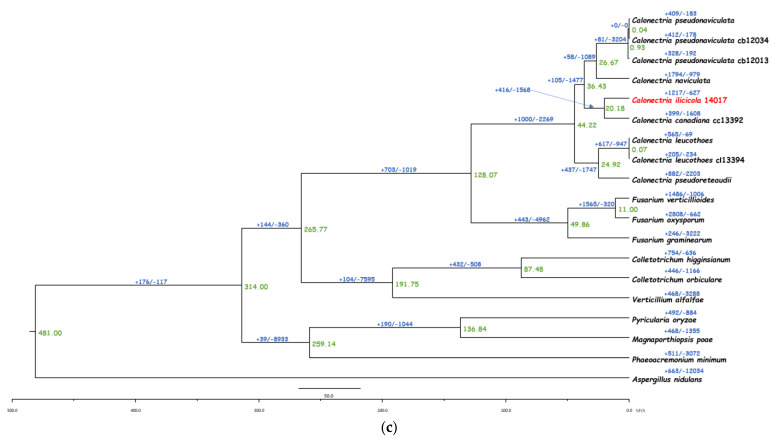
Comparative genomics analysis of *Ci*14017. (**a**) Collinearity map of *Ci*14017 and *Fusarium graminearum* genomes. On the top is the *F. graminearum* genomes, and on the bottom is *Ci*14017, with secretory proteins marked in the red line. (**b**) *Ci*14017 hyperchronic evolution tree. The green numbers represent fossil ages. The blue numbers represent gene family expansion and contraction. The arrow in the figure points to the branch where the bootstrap support rate is located. (**c**) Venn analysis of gene families of 18 different genomes. The values of the black vertical bar chart indicated the number of homologous genes (including the specific genes of five genomes) in one to five genomes, while the values of the yellow horizontal bar chart indicate the total number of homologous genes in one genome with the other four genomes. The red dots represent the homologous genes of the five genomes. The blue dots represent the specific gene of *Ci*14017 and the green dots represent the homologous gene of *C. canadiana cc*13392.

Genomic collinearity analysis revealed that there were up to 31,678 genes in 1993 scaffolds of *Ci*14017 and seven chromosomes of *F. graminearum* strains, with 12,096 genes showing collinearity (38.18%). The 6052 collinear genes in the *Ci*14017 genome were distributed in 270 blocks, whereas the 6044 collinear genes in *F. graminearum* were located in 151 blocks with more than 10 collinearity gene pairs and five blocks with more than 100 collinearity gene pairs (Figure 3a).

Gene family analysis was performed using *F. graminearum* as a model to establish a time-sequence evolutionary tree, which included eight genomes of the same *Calonectria* genus, three genomes of the same Nectriaceae family, six genomes of the Sordariomycetes class, and one genome of the same phylum of Ascomycota. The results showed that *Ci*14017 was closest to *C. canadiana cc*13392. The time of differentiation was estimated at 20.18 million years ago. The number of *Ci*14017 gene families expanded by 1217 and contracted by 627 (Figure 3b).

The *Ci*14017 genome was additionally compared with the genomes of five *Calonectria* species, including *C. leucothoes*, *C. naviculata*, *C. canadiana cc*13392, *C. pseudoreteaudii*, and *C. pseudonaviculata*. Using *F. graminearum* as a model, a Venn analysis of the gene family revealed 8864 core protein-coding genes and 135 genes specific to the *Ci*14017 genome (Figure 3c).

### 3.5. Identification of Effectors

A comprehensive assessment strategy was used to predict secretory proteins with signal peptides, transmembrane domains, extracellular subcellular localization, and exocrine function. Among the 18,366 proteins predicted in the whole genome of *Ci*14017, 1992 were found to possess putative signal peptides at the N-terminal based on SignalP v 4.1 analysis. Subsequent transmembrane domain prediction using TMHMM v 2.0 revealed that 1849 proteins had at least two transmembrane domains. The subcellular localization of 1804 proteins were extracellular. After excluding 86 non-exocrine proteins, 1718 proteins were eventually identified as secreted.

**Figure 4:**
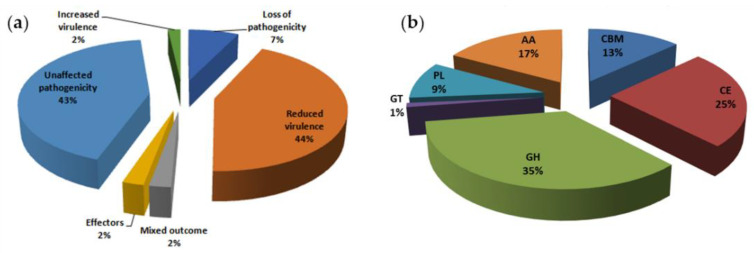
Pathogenic gene analysis of *Ci*14017 effector protein. (**a**) PHI-base comparison results of 289 effector proteins. The text in the figure represents the phenotype after deletion of the mutant gene; (**b**) CAZymes classification of 1718 secreted proteins. CBMs: carbohydrate-binding modules, CEs: carbohydrate esterases, GHs: glucoside hydrolases, GTs: glycosyl transferases, PLs: polysaccharide lyases, AAs: auxiliary activities.

The 1718 proteins harbored 836 protein sequences with cysteine content ≥6, of which 289 with multiple tandem repeats had ≥9. Effector proteins are critical elements for evaluating pathogenicity. Potential effector proteins were screened based on annotation in the PHI-base. Of the 289 proteins, 264 were annotated in the PHI database. Sixteen of these were encoded by genes whose knockdown or knockout would lead to pathogen death, 96 were encoded by hypervirulence genes (gene silencing causes reduced pathogenicity), five were effectors, and five were associated with mixed outcomes. In total, 122 proteins were selected as putative effectors in *Ci*14017 (Figure 4a).

According to the results of the BLAST comparison, among the 1718 secretory proteins, 253 were not found in the NR database. Among 122 candidate effector proteins, 16 had no homologous genes in the NR database. Among the 122 putative genes, 78 had a cysteine content greater than 2%, and 25 had a cysteine content greater than 4%, with the highest being 12.1%. The length of the signal peptide ranged from 14 to 37 aa, and the protein with a signal peptide length of 19 aa had the highest secretory content.

### 3.6. CAZyme Analysis

A total of 482 CAZymes were found among the 1718 secretory proteins and subjected to a CAZyme homology search using the CAZymes analysis toolkit (CAT). The predicted CAZyme distribution is summarized as follows, according to respective classes and gene counts: 82 auxiliary activity, 119 carbohydrated esterases, 168 glycoside hydrolases, five glycosyl transferases, 63 carbohydrate-binding modules, and 45 polysaccharide lyases (Figure 4b). The 63 genes of CBM were classified into 17 gene families; the 119 genes of CE were classified into eight gene families; the 168 genes of GH were classified into 48 gene families; GT genes were classified into four gene families; PL genes were classified into six families; and the 82 genes of AA were classified into seven families. We observed that CE10 (45) CAZymes were most abundant in *Ci*14017, followed by AA7 (39), CE5 (22), GH3 (19), GH43 (19), and PL1 (19). These enzymes are involved in the hydrolysis of carbohydrate and non-carbohydrate substrates (CEs and GHs) as well as in the oxidative degradation of lignin-based components of the plant cell wall (AA).

### 3.7. Identification of 46 Virulence-Associated Factors in C. ilicicola

**Figure 5:**
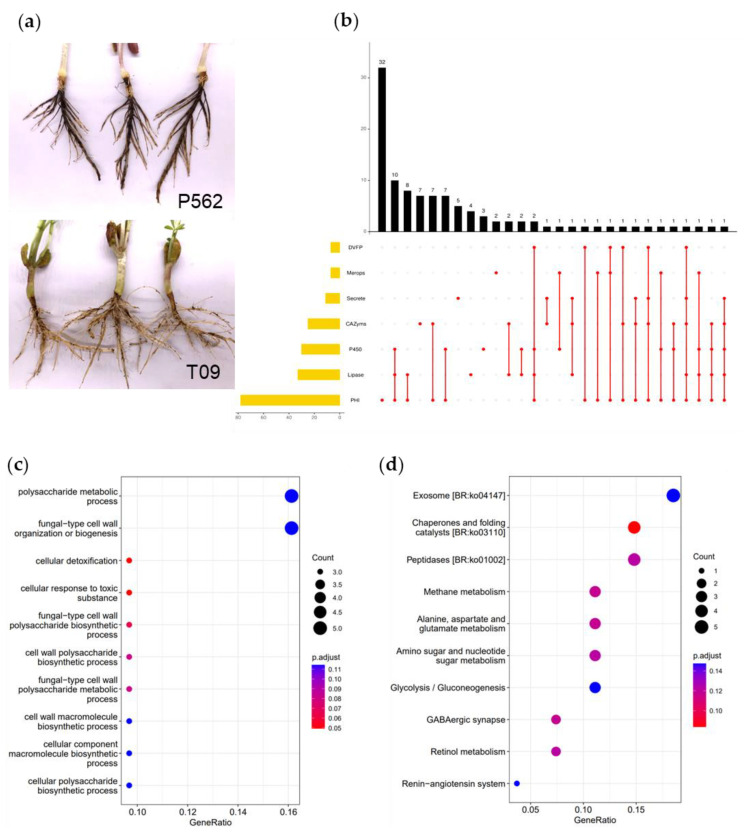
Identification of 46 differentially expressed factor for optimal CBR development. (**a**) Mediate resistance traits of peanut T09 to *Ci*14017. (**b**) Venn analysis of 1146 genes matching the virulence-associated database. The values of the black vertical bar chart indicated the number of matching genes in one to seven databases, while the values of the yellow horizontal bar chart indicate the total number of matching genes in one database. The dots represent the genes that match the which database. (**c**) GO enrichment statistics of 46 proteins. Blast2GO (http://www.geneontology.org/), accessed on 5 May 2022 for GO term annotation. (**d**) KEGG pathway enrichment statistics of 46 proteins. KEGG pathway mapping (http://www.genome.jp/kegg/), accessed on 5 May 2022.

The peanut T09 and P562 cultivars were simultaneously inoculated with *Ci*14017. When compared to the highly sensitive P562, T09 showed moderate resistance to *C. ilicicola.* At 9 days post-inoculation, P562 roots exhibited a severe dark brown and necrotic rot, whereas T09 symptoms were notably lesser, with some visible necrotic rots (Figure 5a). The roots of both T09 and P562 plants were collected for RNA-seq and label-free proteomic analysis through comparison with *C. ilicicola* samples cultured in a PDA medium.

The number of DEGs in P562 and T09 after inoculation with *Ci*14017 (PC/TC) was 5724, with 2464 upregulated genes and 3260 downregulated genes (fold change > 2) (Table S2). There were 536 DEPs in the PC/TC proteome, all of which were upregulated (Table S3).

A total of 7188 expressed genes were identified via transcriptomic analysis. Between these and the 536 upregulated proteins identified via proteomic analysis were 517 overlapping factors. According to proteomic data, another 788 proteins were expressed only in susceptible lines. Of these, 629 overlapped with the 7188 expressed genes. Further, there were 1146 co-differentially expressed genes/proteins between the transcriptome and proteome.

We performed homology searches using the PHI, DFVF, MEROPS, LIPABASE, Cytochrome P450, CAZymes database, and *Ci*14017 secreted proteins in order to identify potential virulence factors. Among the 1146 DEPs, 46 were identified as putative virulence-associated genes (Figure 5b). BLASTP comparison between 46 virulence-associated genes and the NR database showed homologous genes for all candidates.

GO enrichment analysis was conducted for the 46 virulence-associated genes, and the results revealed 804 enriched GO terms (Figure 5c). Genes were mainly enriched in the polysaccharide metabolic process (5 genes) and fungal-type cell wall organization or biogenesis (5 genes). Based on GO enrichment, it can be concluded that the infection with *C. ilicicola* leads to destruction of the peanut cell wall and intercellular structure, resulting in peanut cell decline and death. The 46 virulence-associated genes were enriched in 59 KEGG pathways, as determined via KEGG enrichment analysis (Figure 5d). Genes were mainly enriched in exosomes (five genes), chaperones and folding catalysts (four genes), and peptidases (four genes).

### 3.8. Sequence Analysis of the 46 Virulence-Associated Factors

**Figure 6:**
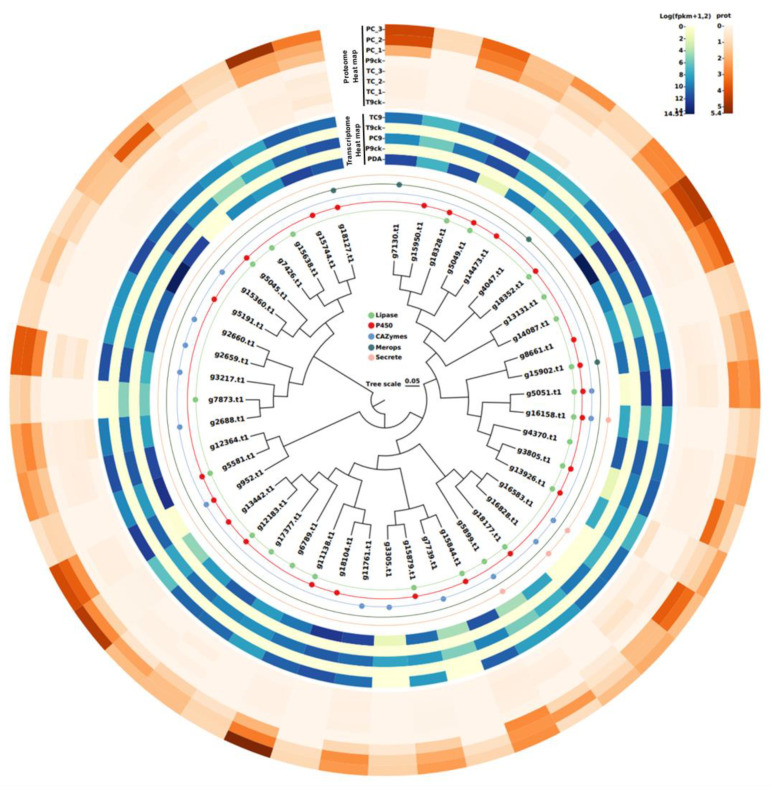
Annotation and expression of 46 putative virulence-associated genes. ML tree of 46 putative virulence-associated genes. The different colors of the round spots indicate the matching results with each database. Transcriptome level gene expression calorimetry. P9ck: P562 9 days after water inoculation; PC9: P562 9 days after *Ci*14017 inoculation; T9ck: T09 9 days after water inoculation; TC9: T09 9 days after *Ci*14017 inoculation; PDA culture conditions. Proteome level gene expression calorimetry. PC_1, PC_2, PC_3: P562 9 days after *Ci*14017 inoculation; P9ck: P562 9 days after water inoculation; TC_1, TC_2, TC_3: T09 9 days after *Ci*14017 inoculation; T9ck: T09 9 days after water inoculation.

The 46 virulence-associated genes were used to construct a phylogenetic tree using RaxML. The outer circles indicate the matching results for each database. There were two merops proteins, eight carbohydrate active enzymes, seven cytochrome P450 enzymes, eight lipases, 20 multi-annotated proteins (Lipase/P450:12; Lipase/P450/Merops:1; Lipase/P450/CAZymes/Secrete:1; CAZymes/Secrete:2; P450/Merops:1; Lipase/P450/CAZymes:1; Lipase/CAZymes/Secrete:1; and P450/CAZymes:1), and one unannotated protein (Figure 6). The gene and protein expression levels of 46 virulence-associated genes were analyzed, revealing expression levels in susceptible lines compared to resistant lines at both levels. At the proteomic level, these 46 virulence-associated genes were not expressed in resistant lines, but were highly expressed in susceptible lines (Figure 6 and Table 2).

**Figure 7:**
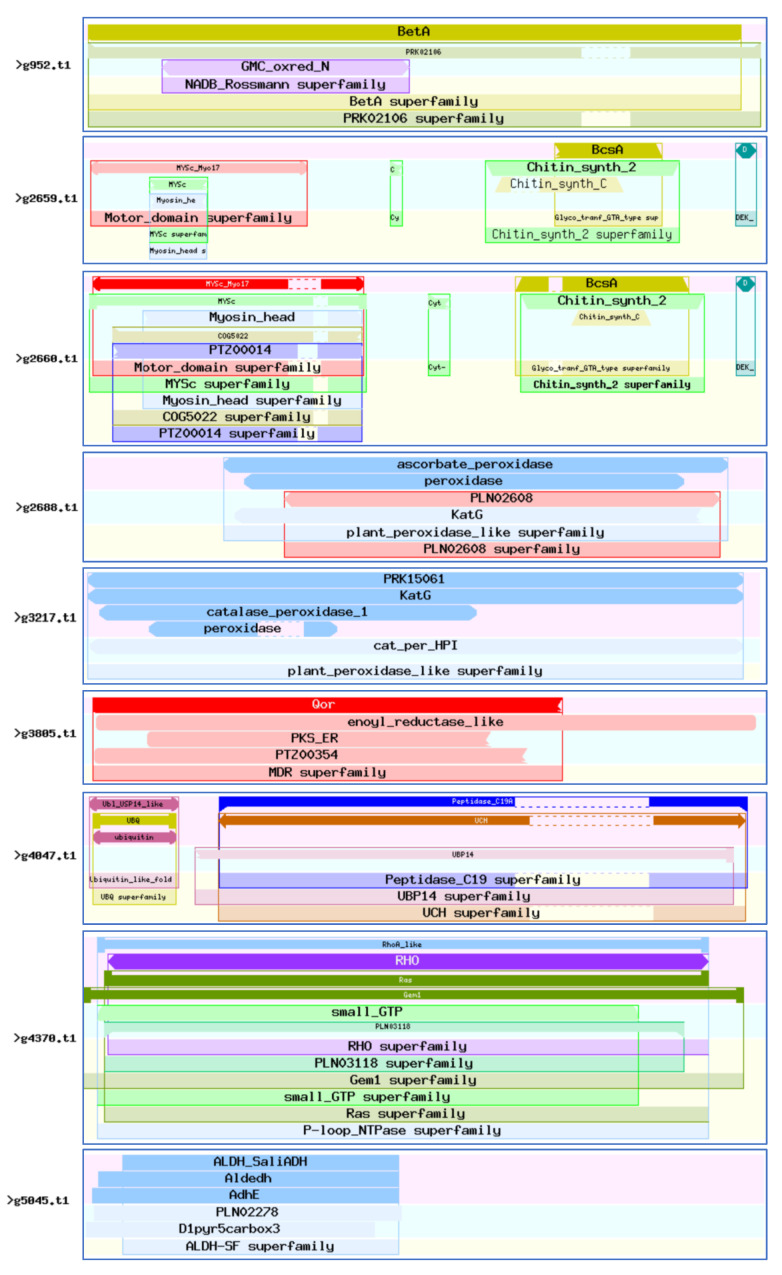

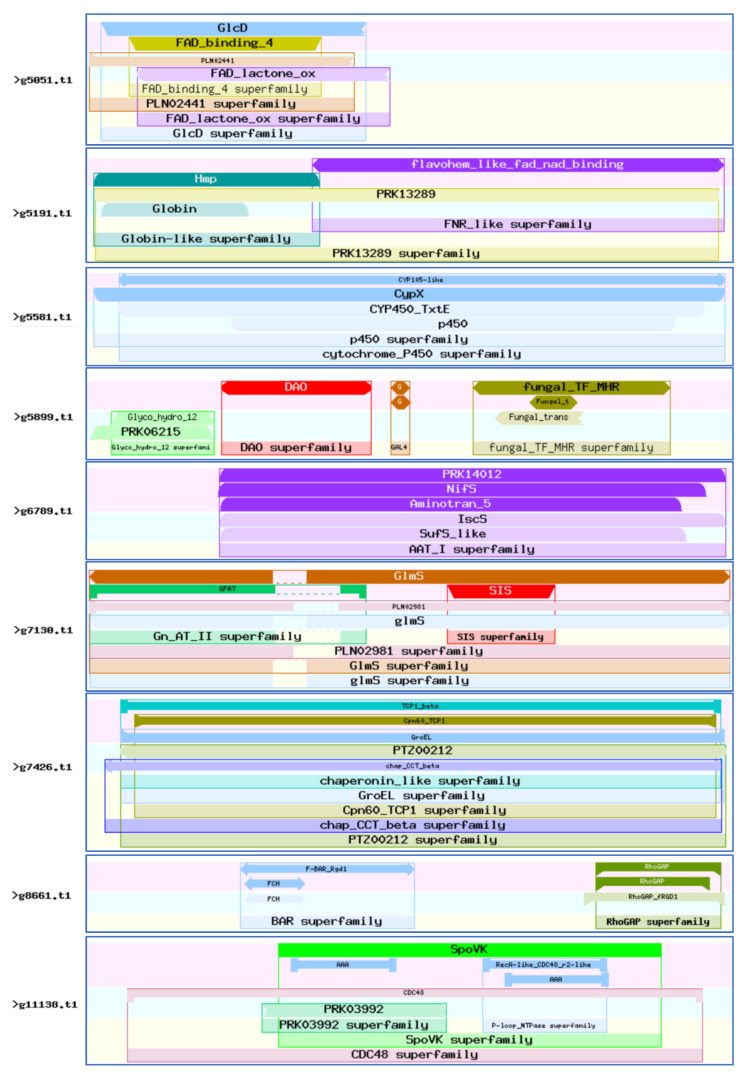

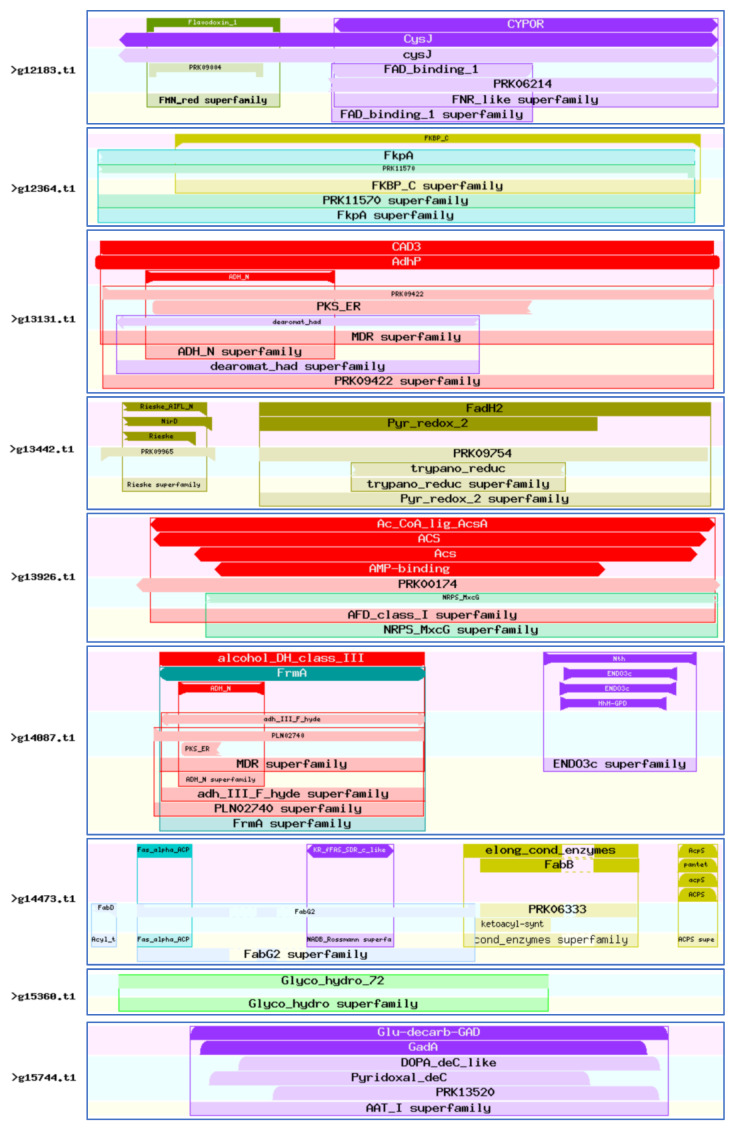

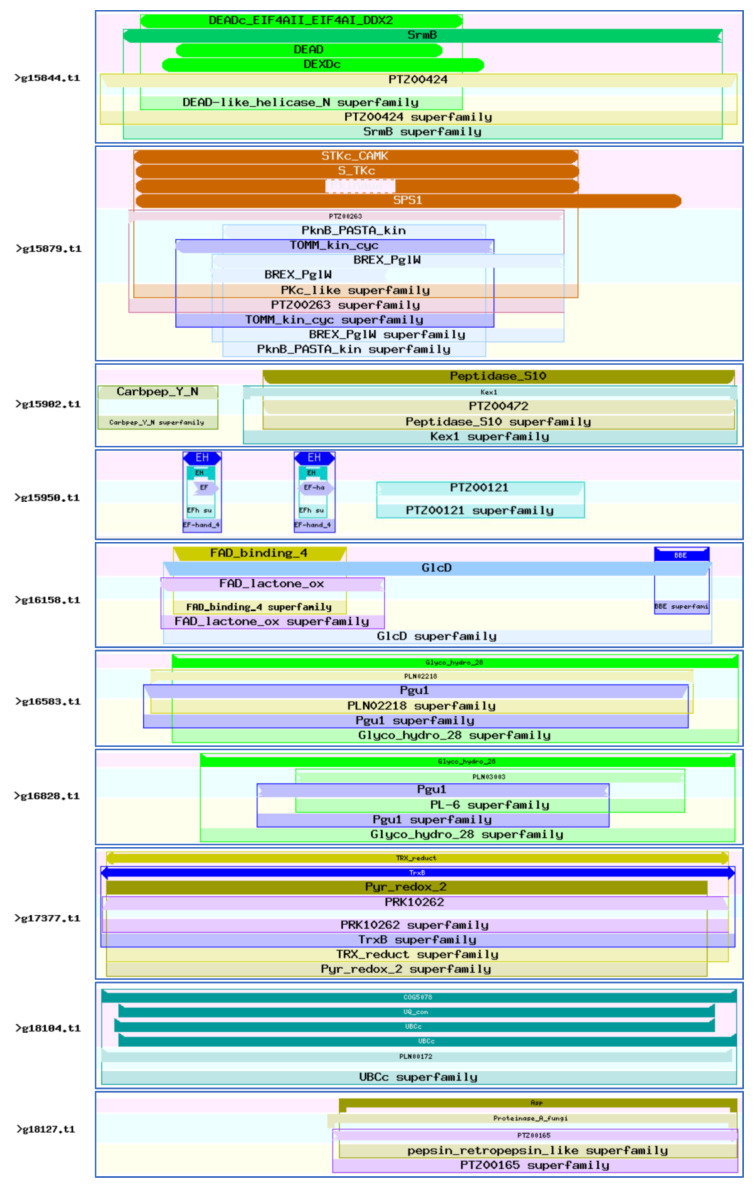

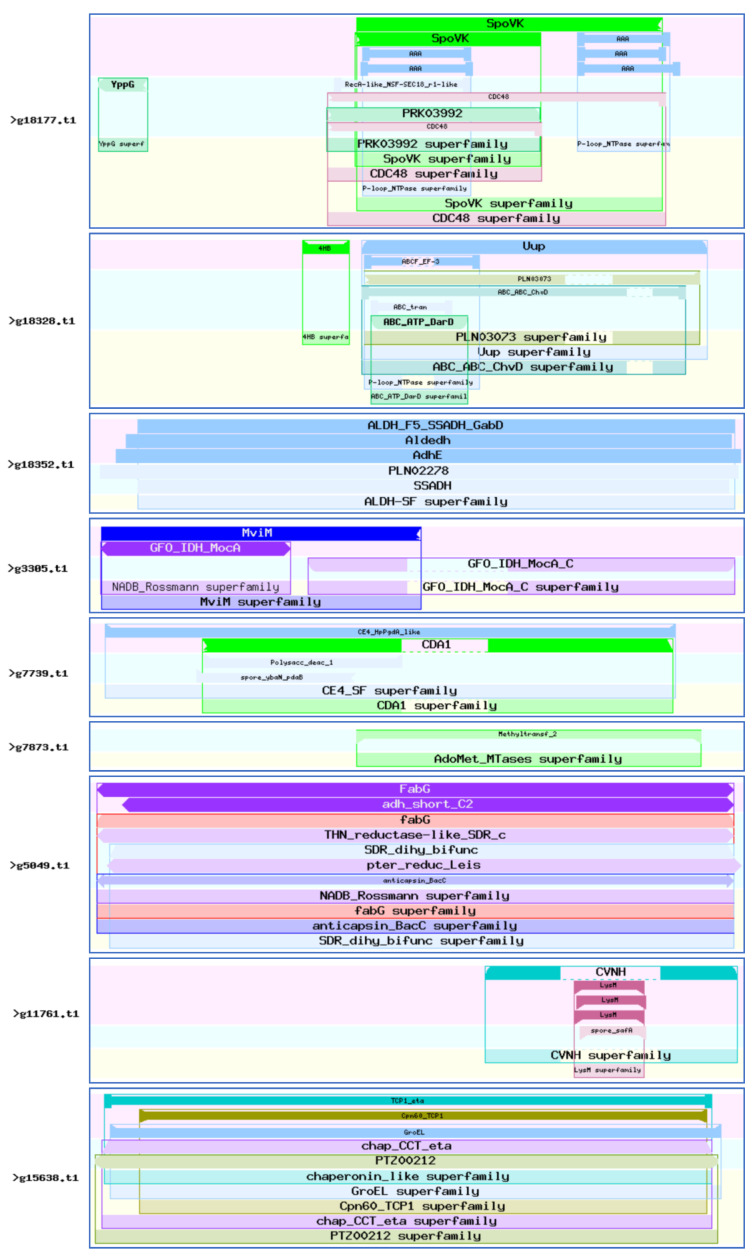
Abundant conserved protein domain. Analysis of 46 virulence-associated genes domains. (https://www.ncbi.nlm.nih.gov/Structure/bwrpsb/bwrpsb.cgi?), accessed on 6 May 2022.

CDD (Conserved Domain Database) is a protein annotation resource consisting of a series of fully annotated domain and full-length protein sequence alignment models. The 46 virulence-associated genes were compared with the CDD database (E-value = 1 × 10^−5^). These genes harbored a total of 2062 CDD protein structural functional domains, 504 clusters, 111 specific features, and 19 generic features. There were six genes with more than 100 protein structural functional domains, among which g18328.t1 contained 210 protein structural functional domains and g4249.t1 contained 136 protein structural functional domains (Figure 7). These proteins are mainly related to the functions of CYP450, such as g5581.t1; ubiquitin, such as g4047.t1 and g18104.t1; chitin synthase, such as g2659.t1 and g2660.t1; fungal-specific transcription factors, such as g5899.t1; and cell division, such as g11138.t1.

## 4. Discussion

In this study, we isolated and characterized *C. ilicicola Ci*14017, which causes peanut black rot in Guangdong Province, China. To screen for candidate pathogenicity factors, we performed whole-genome sequencing with a sequencing depth of 75.84-fold. After reading 1993 scaffolds, the assembled genome size was 66,310,315 bp, coding for 18,366 genes. Based on the assessment of BUSCO profiles for the fungi core database, the matching rate of *Ci*14017 was up to 99%, implying a high-quality genome sequencing performance.

Effectors secreted by plant fungi play critical roles in virulence, as they modulate host defense reactions upon entry into host cells. The particular characteristics of these effectors have been summarized and successfully considered for the identification of putative effectors in a range of plant fungi. Herein, we screened for potential virulence factors according to the following criteria: annotated by PHI-base, associated with reduced virulence, loss of pathogenicity, lethal, or hypervirulence status as described by PHI-base, and localized in the extracellular space. In light of these traits, 1718 secreted proteins were annotated from the *Ci*14017 genome using the MEROPS, DFVF, LIPABASE, CYP450, PHI, and CAZymes databases. This demonstrates that *Ci*14017 harbors effectors similar to those of other known fungi.

The draft and complete genomes of *Calonectria* species have been continuously expanded in recent years. The number of coding genes in *C. leucothoes*, *C. Naviculata*, *C. pseudoreteaudii*, and *C. Pseudonaviculata* varied from 12,964 to 22,409. The number of coding genes in *C. pseudonaviculata cb*12013, *C. pseudonaviculata cb*12034, *C. canadiana cc*13392, and *C. leucothoes cl*13394 obtained in our laboratory in cooperation with Professor Tom Hsiang’s team from the University of Guelph, Canada, ranged from 12,979 to 17,552. Liu et al. estimated 17,308 putative protein-coding sequences in *C. ilicicola* F018 collected in Taiwan [47]. However, the *C. ilicicola* F018 genome was assembled from only 16 contigs. As determined via high-quality genome sequencing, *Ci*14017 contains 18,366 coding genes, a number that falls in the middle among previously reported *Calonectria* species. In addition to the 135 specific proteins in *Ci*14017, 6052 genes in *Ci*14017 were found to overlap with those of *F. graminearum*. Whole-genome evolutionary analysis revealed that *Ci*14017 and *C. canadiana cc*13392 differentiation occurred 20.18 million years ago, with *Ci*14017 expanding by 1217 genes and contracting by 627 genes. This suggests surprising genetic diversity among *Calonectria* species.

Morphology, cytology, and physiological biochemistry have been mainly described for the *C. ilicicola–*host interaction. This study reported the gene expression profiles of *Ci*14017 *in planta* in order to screen putative virulence-associated genes involved in CBR development. We found that 2318 genes were induced by interaction with the sensitive P562 cultivar, and 2363 genes were induced by interaction with the resistant cultivar T09. By comparing the whole-genome gene profiles of sensitive cultivar P562 and resistant cultivar T09, we observed that the expression levels of 2464 genes were higher in the sensitive cultivar P562 than in T09. Proteome analysis revealed 536 DEPs, all upregulated in the susceptible cultivar P562, implying that all of these proteins are essential for *Ci*14017 to cause CBR symptoms. Comparative analysis of transcriptome and proteome data revealed 46 virulence-associated genes, including those encoding ubiquitin thiolesterase, chitin synthase, cytochrome P450, and specific transcription factors.

Ubiquitin-proteasome-mediated protein degradation is an important regulatory mechanism involved in fungal cell life and host adaptation [48]. The pathway includes E1 (ubiquitin-activating enzyme), E2 (ubiquitin-conjugating enzyme), and E3 ubiquitin ligases, which transfer ubiquitin to substrates that are then subjected to degradation in the 26S proteasome. E3 functions as the substrate recognition module of the system and is capable of transferring activated ubiquitin to its substrate. A large number of E3s in fungi are responsible for specifying degraded protein substrates [49]. Disruption of the ubiquitin-proteasome pathways in *Gibberella zeae* results in multiple phenotypic alterations, such as mycelial growth, sexual reproduction, and virulence [50]. The ubiquitin thiolesterase enzyme is involved in the ubiquitin-proteasome system, a cellular pathway responsible for the degradation of misfolded and damaged proteins. This emphasizes the importance of the ubiquitin-proteasome pathway for fungal virulence.

Chitin, a biopolymer composed of β-1,4-linked N-acetylglucosamine, is an important component of fungal cell walls and insect exoskeletons [51]. Chitin synthase plays an essential role in fungal cell wall stability, filamentous mycelial development, and pathogenicity [52]. In *Metarhizium acridum*, seven chitin synthase genes play important roles in growth and development, stress resistance, cell wall integrity, and virulence [53]. Chitin synthesis appears to play an equally essential role in *Ci*14017 causing disease in peanut hosts, as chitin synthases g2659.t1 and g2660.t1 of *Ci*14017 were specifically upregulated in the sensitive cultivar P562.

CYP450 is one of the largest heme-containing protein families and is ubiquitous in all living species [54]. CYP450 enzymes play important roles in primary and secondary metabolism as well as xenobiotic biodegradation in eukaryotes [55]. Plant pathogenic fungi possess relatively more CYP450-coding genes compared with other eukaryotes [56]. Most CYP450 enzymes in *F. graminearum* have redundant functions in xenobiotic detoxification and fungal development, as well as fungal reproduction and virulence [57]. Identification of the g5581.t1 (CYP450) gene as a virulence-associated gene in *Ci*14017 suggests the involvement of CYP450 in fungal developmental processes and virulence.

Several virulence-associated genes are enzymes necessary for metabolic processes, such as S-(hydroxymethyl) glutathione dehydrogenase (g14087.t1). To successfully colonize host plants, fungi need to acquire essential nutrients from their hosts in order to meet requirements for hyphal growth or replication. After inoculation of resistant and susceptible cultivars, the pathogen *Ci*14017 replicated more slowly in resistant cultivars, as these exert a suppressive effect on pathogen replication. The enhanced expression of enzymes involved in metabolic processes implies that *Ci*14017 replicated better in P562 than in T09. On the other hand, enhanced expression of these enzymes helped accelerate metabolic processes, providing more energy for *Ci*14017 replication. For example, S–(hydroxymethyl) glutathione dehydrogenase *MoSFA1* mediates nitric oxide (NO) metabolism by specifically catalyzing the reduction of S–nitrosoglutathione (GSNO), which is involved in conidiation and full virulence of the rice blast fungus *Magnaporthe oryzae* [58].

## 5. Conclusions

We isolated and identified a *C. ilicicola Ci*14017 strain that causes CBR in peanuts. The high-quality whole genome of *C. ilicicola Ci*14017 was obtained by assembling 1993 scaffolds containing 18,366 coding genes. Based on the deciphered genome information, 46 virulence-associated genes were identified through comparison of the transcriptome and proteome between resistant and susceptible peanut cultivars. To our knowledge, this is the first report on virulence-associated genes in *C. ilicicola*, which will help us to further understand the mechanisms through which this fungus causes CBR in host plants.

## Figures and Tables

**Table 1:** Basic information of *Ci*14017 whole genome.

*Ci*14017	Statistical Data
The number of scaffolds	1993
Assembly size (bp)	66,310,315
Mean length (bp)	33,272
Max length (bp)	1,935,007
Min length (bp)	121
Largest 10 total (bp)	14,067,989
The number of scaffolds (>1000 bp)	820
Scaffold N50 (bp)	390,247
Number of gaps	442
Total gap length (bp)	244,347
Mean gap length (bp)	553
Percent N’s	0.37%
GC content	48%
Repetitive DNA	1.53%
tRNA	423
rRNA	90
Predicted coding genes	18,366
Total of exons	53,680
Coding region in genome	42.23%
Mean gene length (bp)	3610
Mean coding gene length (bp)	1525
Mean exons per gene	3
Mean exon length (bp)	522

**Table 2:** Forty-six virulence-associated genes’ relevant information.

SeqName	Description	Annotation to Categorize	Transcriptome/Fold Change (PC/TC)	Proteome/Fold Change (PC/TC)	Length
g11138.t1	Cell division control protein 48	Lipase/P450	0.645397155	3.817191977	836
g11761.t1	CVNH domain-containing protein	CAZymes	1.077780219	2.576375314	409
g12183.t1	NADPH-cytochrome P450 reductase	Lipase/P450	0.777423319	2.274784162	690
g12364.t1	Peptidyl-prolyl cis-trans isomerase	/	0.7462787	2.408002106	114
g13131.t1	alcohol dehydrogenase I	Lipase	0.850951116	4.617233843	350
g13442.t1	Apoptosis-inducing factor 1	P450	3.084508684	3.559369202	546
g13926.t1	Acetyl-coenzyme A synthetase	Lipase/P450	2.340024168	2.520666667	704
g14087.t1	S-(hydroxymethyl)glutathione dehydrogenase	Lipase	0.35893699	3.679765396	888
g14473.t1	Fatty acid synthase subunit alpha	P450	1.920882975	2.612333333	1860
g15360.t1	1,3-beta-glucanosyltransferase Gel1	CAZymes	1.406499686	2.461296413	453
g15638.t1	T-complex protein 1 subunit eta	Lipase	0.933011105	2	557
g15744.t1	Glutamate decarboxylase	P450	0.89437205	3.28017126	571
g15844.t1	eIF4A-like protein	Lipase/P450	0.395142551	2.8717477	397
g15879.t1	CAMK/CAMK1/CAMK1-CMK protein kinase	Lipase/P450	1.036268466	2.70942813	370
g15902.t1	Carboxypeptidase Y A	Lipase/P450/Merops	1.822742632	2.263530052	544
g15950.t1	Actin cytoskeleton-regulatory complex protein PAN1	P450	0.418771014	1.666666667	1501
g16158.t1	Glucooligosaccharide oxidase	Lipase/P450/CAZymes/Secrete	0.375885577	2.000333333	518
g16583.t1	endopolygalacturonase 1	CAZymes/Secrete	0.128246495	2.894074074	359
g16828.t1	probable endopolygalacturonase NFIA_008150	CAZymes/Secrete	0.758522499	1.909192825	382
g17377.t1	Thioredoxin reductase	Lipase	0.46674335	2.260225252	321
g18104.t1	ubiquitin-conjugating enzyme E2-16 kDa	P450	0.804791205	1.666666667	146
g18127.t1	vacuolar protease A	P450/Merops	1.864883004	2.301609848	502
g18177.t1	vesicle fusion factor	Lipase/P450	0.489926572	2.182333333	849
g18328.t1	elongation factor 3	Lipase/P450	0.708137425	3.309420682	1055
g18352.t1	succinate-semialdehyde dehydrogenase (NADP+)	Lipase/P450	0.237659648	3.404154863	494
g2659.t1	chitin synthase	CAZymes	1.141364798	1.666666667	1773
g2660.t1	chitin synthase	CAZymes	1.075445163	1.999666667	1857
g2688.t1	Cytochrome c peroxidase, mitochondrial	CAZymes	1.381410476	2.560020346	357
g3217.t1	catalase-peroxidase	CAZymes	0.853030366	2.82071459	762
g3305.t1	NAD-binding Rossmann fold oxidoreductase family protein	CAZymes	262.7	2	440
g3805.t1	GroES-like protein	Lipase/P450	0.450581519	2.705308465	357
g4047.t1	ubiquitin thiolesterase	Merops	1.087713026	2	555
g4370.t1	GTP-binding protein rhoA	Lipase	1.469419616	2.352234637	195
g5045.t1	Vanillin dehydrogenase	Lipase/P450	0.73931864	2.946907653	1021
g5049.t1	related to short chain dehydrogenase	Lipase/P450	0.908852984	2.114715806	258
g5051.t1	related to 6-hydroxy-D-nicotine oxidase	Lipase/P450/CAZymes	0.727663175	2.707425743	461
g5191.t1	nitric oxide dioxygenase	P450	0.177916187	2	420
g5581.t1	Cytochrome P450 55A3	Lipase/P450	0.595411287	1.999666667	404
g5899.t1	fungal specific transcription factor	Lipase/CAZymes/Secrete	0.148888017	2.146453089	1191
g6789.t1	cysteine desulfurase	Lipase	1.597153312	2	504
g7130.t1	glucosamine-fructose-6-phosphate aminotransferase	Merops	0.508395005	3.672040022	702
g7426.t1	t-complex protein 1 subunit beta	Lipase	0.053649734	2.256364713	540
g7739.t1	Peptidoglycan deacetylase	CAZymes	43.97	1.666666667	335
g7873.t1	S-adenosyl-L-methionine-dependent methyltransferase	Lipase	37.26	2	397
g8661.t1	predicted protein	P450	0.499370829	1.999666667	886
g952.t1	alcohol oxidase	P450/CAZymes	0.340802231	3.471797575	688

## Data Availability

The raw genome data of *Ci*14017 have been deposited in the NCBI with the accession number of SRR19913774. The raw RNA-seq data of the four peanut samples and one *Ci*14017 sample (hyphae) have been deposited in the NCBI with the accession number of SRR19978308, SRR19978307, SRR19977118, SRR19977117. The raw label free data of the twelve peanut samples have been deposited in the PRIDE with the accession number of PXD024578 (private data).

## Supplementary Materials

The following supporting information can be downloaded at: https://www.mdpi.com/article/10.3390/jof8080869/s1, Table S1: Comparison of whole genome assembly results of *Ci*14017 strains; Table S2: *Ci*14017 differentially expressed gene statistics (DEGs); Table S3: *Ci*14017 differentially expressed protein statistics (DEPs).
